# Evaluation of early C‐reactive protein kinetics as an early response marker in biliary tract cancer treated with combined chemoimmunotherapy: A multicenter observational study

**DOI:** 10.1002/cncr.70644

**Published:** 2026-10-13

**Authors:** Gabriel Allo, Joshua Leutiger, Catherine Leyh, Christian Möhring, Ann‐Kristin Zöller, Vera Himmelsbach, Gianluca Ciarlo, Maryam Barsch, Christoph Neumann‐Haefelin, Bertram Bengsch, Fabian Finkelmeier, Mohamad Murad, Cennet Sahin, Marie‐Luise Berres, Maria A. Gonzalez‐Carmona, Christoph Roderburg, Arndt Weinmann, Anna Saborowski, Arndt Vogel, Dirk Waldschmidt

**Affiliations:** ^1^ Department for Gastroenterology and Hepatology, Faculty of Medicine University Hospital of Cologne University of Cologne Cologne Germany; ^2^ Clinic for Gastroenterology, Hepatology, and Infectious Diseases University Hospital Dusseldorf Dusseldorf Germany; ^3^ Department of Medicine I University Hospital of Bonn Bonn Germany; ^4^ Department of Gastroenterology, Hepatology, Infectious Diseases, and Endocrinology Hannover Medical School Hannover Germany; ^5^ Department of Gastroenterology, Medical Clinic 1 University Hospital Frankfurt Frankfurt am Main Germany; ^6^ Clinic for Internal Medicine II University Hospital Freiburg Freiburg Germany; ^7^ Medical Department III University Hospital, Rhenisch‐Westphalian Technical University Aachen Aachen Germany; ^8^ First Department of Medicine University Medical Center of the Johannes Gutenberg University Mainz Germany; ^9^ Division of Hepatology Toronto General Hospital University Health Network Toronto Ontario Canada; ^10^ Division of Gastrointestinal Oncology Princess Margeret Cancer Center Toronto Ontario Canada

**Keywords:** biliary tract neoplasms, C‐reactive protein, cholangiocarcinoma, durvalumab, immune checkpoint inhibitors

## Abstract

**Background:**

Reliable prognostic biomarkers for treatment response in patients with biliary tract cancer (BTC) who receive chemoimmunotherapy are still lacking. The objective of this study was to evaluate the potential of early C‐reactive protein (CRP) kinetics as a tool for predicting survival in these patients.

**Methods:**

The authors retrospectively analyzed patients with BTC who received chemoimmunotherapy at eight tertiary centers and evaluated CRP kinetic models with regard of their prognostic ability within the first 3 months of treatment.

**Results:**

Progression‐free survival (PFS) and overall survival (OS) differed according to CRP dynamics. The median PFS was *not reached* (95% confidence interval [CI], not reached to not reached) for patients with consistently normal CRP, 11.0 months (95% CI, 6.7–15.3 months) for those with initially normal CRP, 6.0 months (95% CI, 3.6–8.4 months) for those with subsequent CRP normalization, and 2.0 months (95% CI, 0.9–3.1 months) for those with persistently elevated CRP. The corresponding median OS was not reached (95% CI, not reached to not reached) for patients with consistently normal CRP, 26 months (95% CI,15.5–36.5 months) for those with initially normal CRP, 23 months (95% CI, 12.0–34.0 months) for those with subsequent normalization, and 9.0 months (95% CI, 6.3–11.7 months) for those with persistently elevated CRP (PFS, log‐rank *p* < .001; OS, *p* = .005). The overall response rates were 50%, 33.3%, 36.0%, and 21.2%, respectively. The prognostic performance of this model remained stable when subgroup definitions were based on 6‐week cutoffs.

**Conclusions:**

CRP kinetics may be a valuable prognostic tool for early response assessment in patients with BTC who receive chemoimmunotherapy. However, these findings require prospective external validation in larger cohorts.

## Abstract 2

In patients with biliary tract cancer who received chemoimmunotherapy, early C‐reactive protein (CRP) normalization or normal CRP values were associated with survival. Assessment of CRP kinetics within the first 6 weeks may provide an inexpensive and simple tool for early prognostic risk stratification, although prospective validation is warranted.

## INTRODUCTION

Biliary tract cancers (BTCs) are a heterogenous group of tumors that are associated with an aggressive nature. Depending on their origin, they are divided into intrahepatic and extrahepatic cholangiocarcinoma and gallbladder carcinoma. Surgical removal is still the only curative option, however, relevant numbers of patients suffer a recurrence, resulting in 5‐year survival rates of only 10‐%–5%.^1^, ^2^ In most patients, the initial diagnosis is not made until advanced, inoperable stages because of a lack of warning symptoms. Therefore, systemic therapy remains the only treatment option in most patients.^3^, ^4^ Until 2022, the combination of the chemotherapeutic agents gemcitabine and cisplatin was the established first‐line therapy, which was associated with a median overall survival (OS) of 11.7 versus 8.1 months with gemcitabine alone.^5^ The addition of the programmed death‐ligand 1 (PD‐L1) inhibitor durvalumab let to a significantly longer OS in first‐line treatment and is now considered the standard of care in BTC.^6^ In the follow‐up analysis of the TOPAZ‐1 study, a 24‐month OS rate of 23.6% versus 11.5% was demonstrated.^7^ Recently, a survival benefit was also demonstrated for the addition of the anti‐programmed death 1 (PD‐1) monoclonal antibody pembrolizumab, thus underlining the additive effects of the combination of immune checkpoint inhibition (ICI) and chemotherapy.^8^

Nevertheless, many patients do not respond to ICI; and, to date, there are no biomarkers that reliably predict treatment response in BTC. High tumor mutation burden and a tumor area positivity score for PD‐L1 are established prognostic biomarkers in ICI treatment across many tumor entities, but their prognostic ability is limited in BTC.^6^, ^8^, ^9^, ^10^ Microsatellite instability, which is associated with an improved response to ICI, accounts only for about 1%–3% of patients with BTC,^11^, ^12^ limiting its use in the general BTC population. However, because a relevant proportion of patients with biliary tumors are candidates for promising second‐line targeted therapy, early detection of a nonresponse to chemoimmunotherapy is of high relevance.^13^, ^14^, ^15^

Recent studies have demonstrated the prognostic value of inflammatory markers with regard to the response to ICI therapy.^16^ In particular, early C‐reactive protein (CRP) kinetics after the initiation of ICI therapy was shown to have a relevant prognostic value. Fukuda et al. postulated three groups of CRP kinetics: *CRP flare responders*, who had a doubling of CRP values within the first 4 weeks after ICI initiation followed by a decline below baseline within the first 3 months; *CRP responders*, who had a decrease in CRP values of at least 30% within the first 3 months; and *CRP nonresponders*, who did not meet the definition of the other two groups.^17^ This model was able to demonstrate its prognostic value in various tumor entities.^18^, ^19^, ^20^, ^21^, ^22^

Ishihara et al. proposed an alternative model that distinguishes between patients who have normal CRP values at ICI initiation and two additional groups with initially elevated CRP levels—those whose CRP levels normalize during treatment and those whose CRP levels remain elevated.^23^ This model was developed in a cohort of patients with metastatic renal cell carcinoma and lacks validation in other tumor entities. Notably, none of these models have been validated in cohorts treated with chemoimmunotherapy. Therefore, the objective of the current study was to test the prognostic ability of early CRP kinetics in patients with BTC undergoing chemoimmunotherapy.

## MATERIALS AND METHODS

### Patients

This multicenter national observational study was conducted at eight medical centers in Germany. Inclusion criteria were age over 18 years, presence of BTC (intrahepatic and extrahepatic cholangiocarcinoma, papillary carcinoma and gallbladder carcinoma), treatment start with durvalumab between 01/01/2021 and 31/12/2023, the presence of at least one CRP value 30 days before and after the start of ICI as well as another value until the first staging examination or at the time of clinical progression. Exclusion criteria were the use of a local ablative, radiotherapeutic or surgical procedure within 10 days before and in the first four weeks after the start of ICI as well as an acute infectious disease within the first four weeks after the start of ICI. Cases with insufficient longitudinal CRP measurement to characterize CRP groups were excluded (complete‐case analysis). Missing CRP data were not imputed. A flow chart of the study population is shown in Figure S1.

### Treatment

In accordance with the TOPAZ‐1 study, patients received durvalumab (1500 mg) intravenously on a 21‐day cycle for up to eight cycles. In addition, on day 1s and 8, patients received gemcitabine (1000 mg/m^2^) and cisplatin (25 mg/m^2^). After eight cycles, patients received durvalumab monotherapy every 4 weeks until clinical or radiologic disease progression.^6^ Treatment applications or dose modifications were defined by local standards.

### Variables and end points

The relevant patient baseline characteristics and parameters, which were retrieved from the electronic medical records, included: sex, age, tumor subtype, greatest tumor dimension, Eastern Cooperative Oncology Group (ECOG) performance status, tumor stage, baseline albumin, carbohydrate antigen 19‐9 (CA 19‐9), neutrophil‐to‐lymphocyte ratio (NLR), bilirubin, CRP values, and the occurrence of infections. Radiologic response was evaluated by magnetic resonance imaging or computed tomography in analogy to RECIST (Response Evaluation Criteria in Solid Tumors), version 1.1, by local radiologists.^24^

Progression‐free survival (PFS), OS, the disease control rate (DCR), and the objective response rate (ORR) were co‐primary end points. PFS was defined as the time from ICI initiation until progression or death. OS was defined as the time from ICI initiation to death from any cause. The ORR was defined as the percentage of patients with radiologic complete or partial remission. The DCR was defined as the percentage of patients with at least stable disease in staging imaging.

### Definitions of CRP models

Patients were classified into the three CRP‐response groups previously defined by Fukuda et al.^17^ CRP flare responders were defined as patients who had a two‐fold increase in baseline CRP values within the first 4 weeks after the first durvalumab application, followed by a drop below baseline within the 3 months after treatment initiation. Patients with a decrease in CRP levels ≥30% below baseline within the first 3 months of treatment were defined as CRP responders. All other patients were classified as CRP nonresponders in this model. For the CRP kinetics model of Ishihara et al., patients with CRP levels ≤5 mg/L were classified as *CRP normal*, whereas patients with initially elevated CRP levels and a subsequent decline below 5 mg/L during the first 3 months were classified as *normalized*. We selected a CRP cutoff value of ≤5 mg/L, rather than the 10‐mg/L cutoff used in the original publication by Ishihara and colleagues,^23^ because 5 mg/L represents the upper limit of normal (ULN) according to the laboratory reference ranges of the participating medical centers. In a refined model, patients who had CRP levels consistently ≤5 mg/L throughout the first 3 months were included in the *all‐normal* group in reference to Barth et al.^22^ All other patient were classified as *non‐normalized*.

### Statistical analysis

Descriptive analyses were conducted using Statistical Package for the Social Sciences (SPSS), version 29 (IBM Corporation) and R (version 4.5.1; R Foundation for Statistical Computing).

Categorical variables were reported as median, frequencies, ranges, and interquartile ranges (IQRs). χ2 tests, Fisher exact tests, and Kruskal–Wallis tests were used to compare groups where applicable. PFS and OS after durvalumab initiation were assessed by Kaplan–Meier analysis and compared using the log‐rank test with censoring at the last known follow‐up if progression or death had not occurred. Univariable and multivariable Cox regression analyses were performed to compare the prognostic value of analyzed variables, which were only included in multivariable analysis if they were significant in univariable models (*p* values < .05). To evaluate whether longitudinal CRP kinetics could provide prognostic information beyond baseline CRP values, baseline CRP was also included in the univariate and multivariate analyses as a categorical variable (lower vs. higher than median baseline CRP). This threshold was chosen to reduce conceptual overlap and collinearity with the *normal* CRP subgroup of the Ishihara et al. classification, which is defined by baseline CRP levels below the ULN. Because CRP classifications incorporate the first 3 months after ICI initiation, CRP values could only fulfill the normalization or response criteria if patients survived until the respective CRP thresholds were reached. Otherwise, they would be disproportionately assigned to unfavorable groups, such as the *non‐normalized* group. To address this possible immortal time bias, we added landmark analysis after 6 weeks and 3 months. All tests were two sided, and *p* values < .05 were considered significant.

This study was conducted according to the Declaration of Helsinki and was approved by the responsible ethical review board (Ethical Review Board of the University of Cologne, 50931 Cologne: 23‐1466‐retro). Written informed consent to participate was waived because of the strict retrospective design of this study.

An abstract of this work with preliminary results was presented at the American Society of Clinical Oncology Gastrointestinal Cancers Symposium (January 8–10, 2026; San Francisco, California).^25^

## RESULTS

### Baseline characteristics

Iin total, 104 patients with BTC started treatment with durvalumab by December 31, 2023 (Table 1). The median age was 62 years (IQR, 57–66 years), and 46 patients (44.2%) were women. Intrahepatic cholangiocarcinoma was the most common subtype (*n* = 59; 56.7%), and 69 patients (74.1%) had a metastatic tumor stage. During the median follow‐up period of 29 months, 75 patients (72.1%) experienced progression, and 62 (59.6%) died. The DCR and ORR were 62.5% and 29.8%, respectively (Table 1).

**TABLE 1:** Baseline characteristics.

Characteristic	No. (%)
Age: Median [IQR], years	62 [57–66]
Female	46 (44.2)
Subtype of biliary tract cancer	
Intrahepatic cholangiocarcinoma	59 (56.7)
Extrahepatic cholangiocarcinoma	38 (36.5)
Gallbladder carcinoma	7 (6.7)
Greatest lesion dimension [IQR] in cm, *n* = 74	5.0 [2.3–9.0]
Disease stage, *n* = 93	
Metastatic disease	69 (74.1)
Locally advanced	24 (25.8)
Molecular profile *n* = 83	
MSI high	3 (3.6)
FGFR2	7 (8.4)
IDH1	8 (9.6)
History of viral hepatitis	7 (6.7)
ECOG PS, *n* = 102	
0	63 (60.6)
≥1	39 (37.5)
Concurrent steroid medication, *n* = 103	4 (3.8)
Albumin [IQR], g/L	38 [34–42]
Bilirubin [IQR], mg/dL	0.5 [0.3–0.7]
NLR [IQR]	2.8 [1.4–5.1]
CA 19‐9 [IQR], kU/L	67 [17–316]
Baseline CRP [IQR], mg/L	6.8 [2.8–28.4]

Abbreviations: CA 19‐9, carbohydrate antigen 19‐9; CRP, C‐reactive protein; ECOG PS, Eastern Cooperative Oncology Group performance status; FGFR2, fibroblast growth factor receptor 2; IDH1, isocitrate dehydrogenase 1; IQR, interquartile range; MSI, microsatellite instability; NLR, neutrophil‐to‐lymphocyte ratio.

### CRP kinetic model according to Fukuda

According to the model of Fukuda et al., 29 (27.9%), 43 (41.3%), and 32 (30.8%) patients were assigned to the flare responder, responder, and nonresponder groups, respectively. The groups did not differ with regard to patient characteristics such as age, sex, ECOG performance status, tumor subtype, greatest tumor dimension, disease stage, bilirubin level, steroid use during treatment, time to first staging, or CA 19‐9 (see Table S1). However, laboratory values of albumin, NLR, and baseline CRP differed significantly between groups.

The DCR and ORR did not differ significantly between flare responders, responders, and nonresponders. The DCRs were 67.9%, 63.4%, and 64.5% (*p* = .928), respectively, and the ORRs were 25.0%, 34.1%, and 32.3% (*p* = .71), respectively. The median PFS exhibited no significant difference between the groups (*p* = .319) and was 6.0 months (95% CI, 0.9–11.1 months) for flare responders, 8.0 months (95% CI, 5.2–10.8 months) for responders, and 5.0 months (95% CI, 0.4–9.6 months) for nonresponders (Figure 1A). The median OS exhibited no significant differences between the groups (*p* = .333) and was 15.0 months (95% CI, 6.8–23.2 months) for flare responders, 16.0 months (95% CI, 6.3–25.6 months) for responders, and 26.0 months (95% CI, 11.3–40.7 months) for nonresponders (Figure 1B).

**FIGURE 1:**
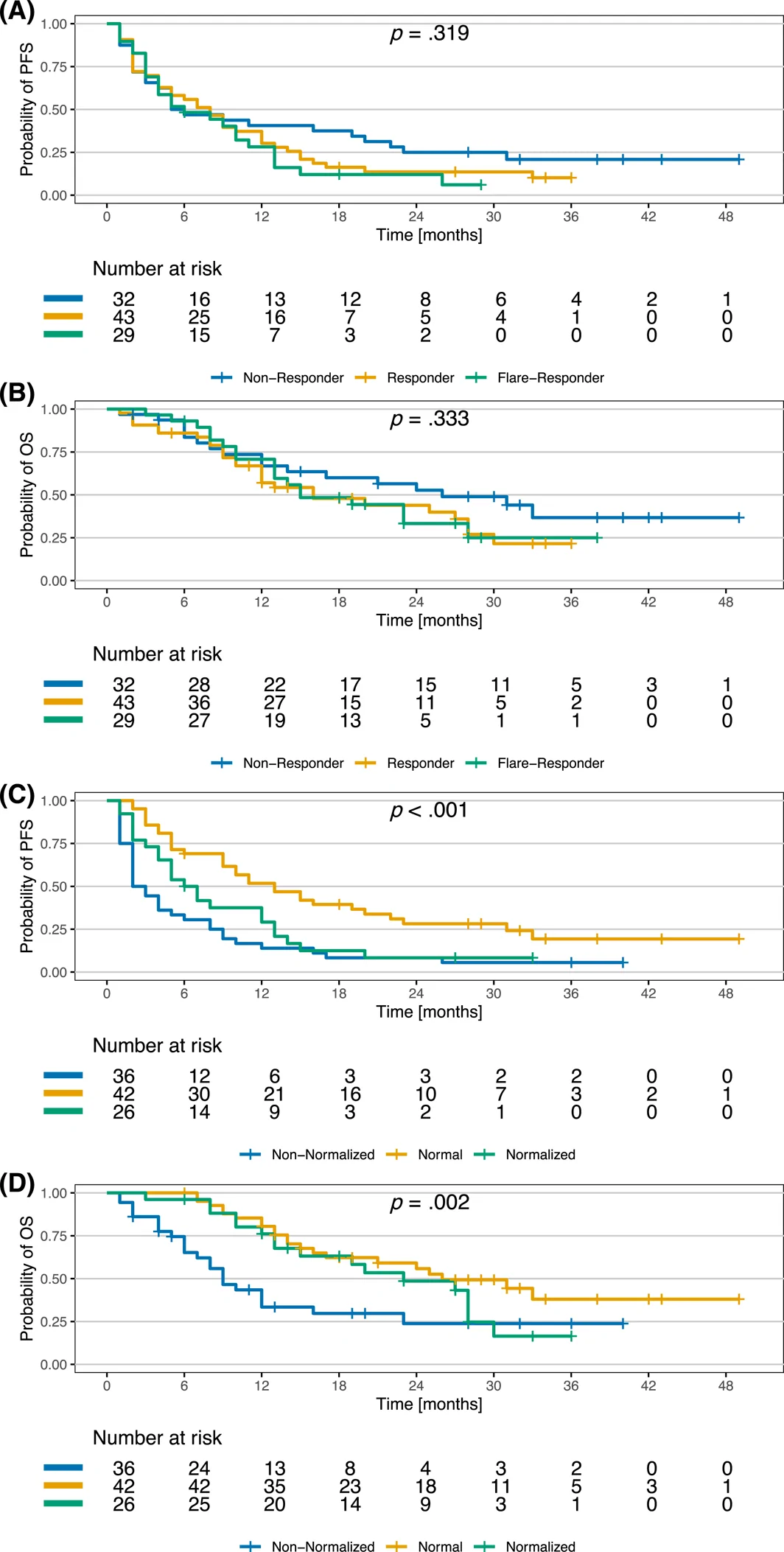
Kaplan–Meier plots for (A,C) progression‐free survival and (B,D) overall survival according to the models of (A,B) Fukuda et al.^17^ and (C,D) Ishihara et al.^23^ OS indicates overall survival; PFS, progression‐free survival.

### CRP kinetics model according to Ishihara

In the study cohort, 42 (40.4%), 26 (25.0%), and 36 (34.6%) patients could be assigned to the CRP‐normal, CRP‐normalized, and CRP–non‐normalized groups, respectively. The groups did not differ in terms of patient characteristics such as age, sex, ECOG performance status, tumor subtype, metastases, bilirubin, time to first staging examination, median time between staging examinations, or steroid use during ICI (see Table S2). However, patients in the CRP‐normal and CRP‐normalized groups had lower NLR and baseline CRP levels, whereas albumin levels and greatest tumor dimensions were higher than in the CRP–non‐normalized group.

The DCR differed significantly (*p* = .012) between the normal, normalized, and non‐normalized groups at 81.0%, 60.0%, and 48.5%, respectively. The normal and normalized groups tended to have a higher ORR (35.7% and 36.0%, respectively) than the non‐normalized group (21.2%), but this difference was not significant (*p* = .332).

The median PFS differed significantly between the groups (*p* < .001) and was 13.0 months (95% CI, 7.9–18.2 months) for the normal group, 6.0 months (95% CI, 3.6–8.4 months) for the normalized group, and 2.0 months (95% CI, 0.9–9.3 months) for the non‐normalized group (Figure 1C).

The median OS also differed significantly between the groups (*p* = .002) and was 26.0 months (95% CI, 16.0–36.1 months) for the normal group, 23 months (95% CI, 12.0–34.0 months) for the normalized group, and 9.0 months (95% CI, 6.3–11.7 months) for the non‐normalized group (Figure 1D).

### Univariable and multivariable regression model

In univariable Cox regression models for PFS and OS, according to Fukuda and colleagues’ model, CRP responders did not have a significantly lower hazard of death than nonresponders (Tables 2 and 3). In contrast, according to the Ishihara et al. model, the CRP normal group had a significantly lower hazard of progression or death than the non‐normalized group, and both the CRP‐normal and CRP‐normalized groups demonstrated higher odds of OS than the CRP non‐normalized group (Tables 2 and 3).

**TABLE 2:** Univariable and multivariable Cox regression models for progression‐free survival for the original and refined model for early C‐reactive protein kinetics.

Variable	Univariate analysis		Multivariate analysis^a^			
			Ishihara original		Ishihara refined	
	HR (95% CI)	*p*	HR (95% CI)	*p*	HR (95% CI)	*p*
Age, years						
≤62	1.00 (reference)					
>62	0.622 (0.731–1.689)	.622				
Sex						
Male	1.00 (reference)	.011^b^	1.00 (reference)	.028^b^	1.00 (reference)	.038^b^
Female	0.572 (0.372–0.882)		0.596 (0.376–0.945)		0.616 (0.390–0.974)	
Subtype of biliary tract cancer		.013^b^		.002^b^		.03^b^
Intrahepatic cholangiocarcinoma	1.00 (reference)		1.00 (reference)		1.00 (reference)	
Extrahepatic cholangiocarcinoma	0.563 (0.355–0.893)	.015^b^	0.526 (0.323–0.855)	.01^b^	0.541 (0.332–0.881)	.013^b^
Gallbladder carcinoma	1.723 (0.724–4.1)	.219	2.644 (1.082–6.464)	.033^b^	2.537 (1.037–6.204)	.041^b^
Distant metastasis						
No	1.00 (reference)	.172				
Yes	1.372 (0.871–2.162)					
ECOG PS						
0	1.00 (reference)	.1				
>1	1.437 (0.932–2.2149					
Concurrent steroid medication						
No	1.00 (reference)	.249				
Yes	1.823 (0.657–5.056)					
Baseline CRP, mg/L						
≤Median	1.00 (reference)	.01^b^	1.00 (reference)	.014^b^	1.00 (reference)	.013^b^
>Median	1.739 (1.140–2.652)		2.916 (1.244–6.832)		2.933 (1.253–6.870)	
CA 19‐9, kU/L						
≤Median	1.00 (reference)	.735				
>Median	1.077 (0.701–1.654)					
NLR						
≤3.0	1.00 (reference)	.229				
>3.0	1.336 (0.834–2.141)					
Albumin, g/L						
≥35	1.00 (reference)	.316				
<35	1.293 (0.783–2.134)					
Bilirubin, mg/dL						
<0.455	1.00 (reference)	.708				
>0.455	1.084 (0.711–1.652)					
Molecular profiling						
MSI						
No	1.00 (reference)	.158				
Yes	0.240 (0.033–1.738)					
IDH1						
No	1.00 (reference)	.754				
Yes	1.134 (0.517–2.485)					
FGFR2						
No	1.00 (reference)	.978				
Yes	1.011 (0.462–2.213)					
BRAF						
No	1.00 (reference)	.978				
Yes	0.984 (0.308–3.147)					
Greatest lesion dimension						
<Median	1.00 (reference)	.469				
≥Median	1.201 (0.732–1.972)					
History of viral hepatitis						
No	1.00 (reference)	.715				
Yes	0.857 (0.373–1.965)					
CRP response: Fukuda^c^		.354				
Nonresponder	1.00 (reference)					
Responder	1.311 (0.787–2.182)	.298				
Flare responder	1.502 (0.852–2.647)	.159				
CRP kinetics: Ishihara^a^		< .001^b^		< .001^b^		
Non‐normalized	1.00 (reference)					
Normal	0.379 (0.231–0.621)	< .001^b^	0.141 (0.051–0.388)	< .001^b^		
Normalized	0.685 (0.403–1.166)	.164	0.447 (0.229–0.872)	.018^b^		
CRP kinetics: Ishihara redefined^a^		.001^b^				.001^b^
Non‐normalized	1.00 (reference)					
Normal	0.433 (0.262–0.716)	.001^b^			0.155 (0.056–0.427)	< .001^b^
Normalized	0.686 (0.403–1.167)	.164			0.447 (0.229–0.872)	.018^b^
All normal	0.133 (0.032–0.555)	.006^b^			0.055 (0.01–0.299)	< .001^b^

Abbreviations: CA 19‐9, carbohydrate antigen 19‐9; CI, confidence interval; CRP, C‐reactive protein; ECOG PS, Eastern Cooperative Oncology Group performance status; FGFR2, fibroblast growth factor receptor 2; HR, hazard ratio; IDH1, isocitrate dehydrogenase 1; MSI, microsatellite instability; NLR, neutrophil‐to‐lymphocyte ratio.

^a^ See: Ishihara H, Takagi T, Kondo T, et al. Predictive impact of an early change in serum C‐reactive protein levels in nivolumab therapy for metastatic renal cell carcinoma. Urol Oncol. 2020;38(5):526‐532.^23^

^b^ These *p* values indicate statistical significance.

^c^ See: Fukuda S, Saito K, Yasuda Y, et al. Impact of C‐reactive protein flare‐response on oncological outcomes in patients with metastatic renal cell carcinoma treated with nivolumab. J Immunother Cancer. 2021;9(2):e001564.^17^

**TABLE 3:** Univariable and multivariable Cox regression models for overall survival for the original and refined model for early C‐reactive protein kinetics.

Variable	Univariate analysis		Multivariate analysis^a^			
			Ishihara original		Ishihara refined	
	HR (95% CI)	*p*	HR (95% CI)	*p*	HR (95% CI)	*p*
Age, years						
≤62	1.00 (reference)					
>62	1.632 (0.988–2.694)	.059				
Sex						
Male	1.00 (reference)					
Female	0.735 (0.441–1.227)	.239				
Subtype of biliary tract cancer		.039^b^		.021^b^		.023^b^
Intrahepatic cholangiocarcinoma	1.00 (reference)		1.00 (reference)		1.00 (reference)	
Extrahepatic cholangiocarcinoma	0.750 (0.431–1.306)	.309	0.763 (0.425–1.367)	.363	0.769 (0.428–1.379)	.378
Gallbladder carcinoma	2.551 (1.051–6.187)	.038^b^	3.122 (1.261–7.73)	.014^b^	3.089 (1.246–7.659)	.015^b^
Distant metastasis						
No	1.00 (reference)					
Yes	1.584 (0.904–2.774)	.108				
ECOG PS						
0	1.00 (reference)	< .001^b^	1.00 (reference)	< .001^b^	1.00 (reference)	< .001^b^
>1	3.065 (1.84–5.1)		2.986 (1.758–5.074)		2.951 (1.732–5.026)	
Concurrent steroid medication						
No	1.00 (reference)					
Yes	1.542 (0.481–4.941)	.466				
Baseline CRP, mg/L						
≤Median	1.00 (reference)		1.00 (reference)		1.00 (reference)	
>Median	1.679 (1.018–2.77)	.043^b^	2.670 (0.984–7.245)	.054	2.660 (0.980–7.213)	.055
CA 19‐9, kU/L						
≤Median	1.00 (reference)					
>Median	0.1525 (0.917–2.535)	.104				
NLR						
≤3.0	1.00 (reference)					
>3.0	1.117 (0.633–1.972)	.703				
Albumin, g/L						
≥35	1.00 (reference)					
<35	1.385 (0.763–2.516)	.284				
Bilirubin, mg/dL						
<Median	1.00 (reference)	.152				
>Median	1.443 (0.873–2.384)					
Molecular profiling						
MSI						
No	1.00 (reference)	.22				
Yes	0.289 (0.4–2.1)					
IDH1						
No	1.00 (reference)	.415				
Yes	0.654 (0.236–1.816)					
FGFR2						
No	1.00 (reference)	.727				
Yes	0.848 (0.337–2.138)					
BRAF						
No	1.00 (reference)	.968				
Yes	1.03 (0.25–4.24)					
Greatest lesion dimension						
<5 cm	1.00 (reference)	.115				
≥5 cm	1.652 (0.885–3.084)					
History of viral hepatitis						
No	1.00 (reference)	.969				
Yes	0.98 (0.355–2.703)					
CRP response: Fukuda^c^		.317				
Nonresponder	1.00 (reference)					
Responder	1.567 (0.849–2.894)	.151				
Flare responder	1.522 (0.775–2.992)	.223				
CRP kinetics: Ishihara^a^		.002^b^		.05^b^		
Non‐normalized	1.00 (reference)					
Normal	0.352 (0.195–0.637)	< .001^b^	0.169 (0.053–0.540)	.03^b^		
Normalized	0.517 (0.276–0.967)	.039^b^	0.285 (0.124–0.657)	.03^b^		
CRP kinetics: Ishihara redefined^a^		.006^b^				.015^b^
Non‐normalized	1.00 (reference)					
Normal	0.377 (0.205–0.692)	.002^b^			0.174 (0.054–0.562)	.003^b^
Normalized	0.517 (0.276–0.967)	.039^b^			0.287 (0.124–0.660)	.003^b^
All normal	0.217 (0.051–0.924)	.039^b^			0.135 (0.023–0.783)	.026^b^

Abbreviations: CA 19‐9, carbohydrate antigen 19‐9; CI, confidence interval; CRP, C‐reactive protein; ECOG PS, Eastern Cooperative Oncology Group performance status; FGFR2, fibroblast growth factor receptor 2; HR, hazard ratio; IDH1, isocitrate dehydrogenase 1; MSI, microsatellite instability; NLR, neutrophil‐to‐lymphocyte ratio.

^a^ See: Ishihara H, Takagi T, Kondo T, et al. Predictive impact of an early change in serum C‐reactive protein levels in nivolumab therapy for metastatic renal cell carcinoma. Urol Oncol. 2020;38(5):526‐532.^23^

^b^ These *p* values indicate statistical significance.

^c^ See: Fukuda S, Saito K, Yasuda Y, et al. Impact of C‐reactive protein flare‐response on oncological outcomes in patients with metastatic renal cell carcinoma treated with nivolumab. J Immunother Cancer. 2021;9(2):e001564.^17^

The association of Ishihara and colleagues’ model with survival was confirmed after multivariable adjustment for sex, baseline CRP, and cancer subtype with regard to PFS and for ECOG performance status, cancer subtype and baseline CRP with regard to OS (Tables 2 and 3). These factors were significantly associated in the univariate analysis.

### Refined model by Ishihara

Consistently low CRP levels (below the ULN) were detected in six patients (5.8%) in the first 3 months after ICI initiation. These patients with all‐normal CRP levels had higher albumin levels than those in the non‐normalized group and had the lowest median NLR, baseline CRP levels (Figure 2), greatest tumor dimensions, more favorable performance status, as well as the highest CA 19‐9 levels at the start of treatment. Other variables did not differ significantly between the groups (Table 4). Reclassifying these patients allowed us to modify the Ishihara et al. CRP kinetic model into a four‐group model.

**FIGURE 2:**
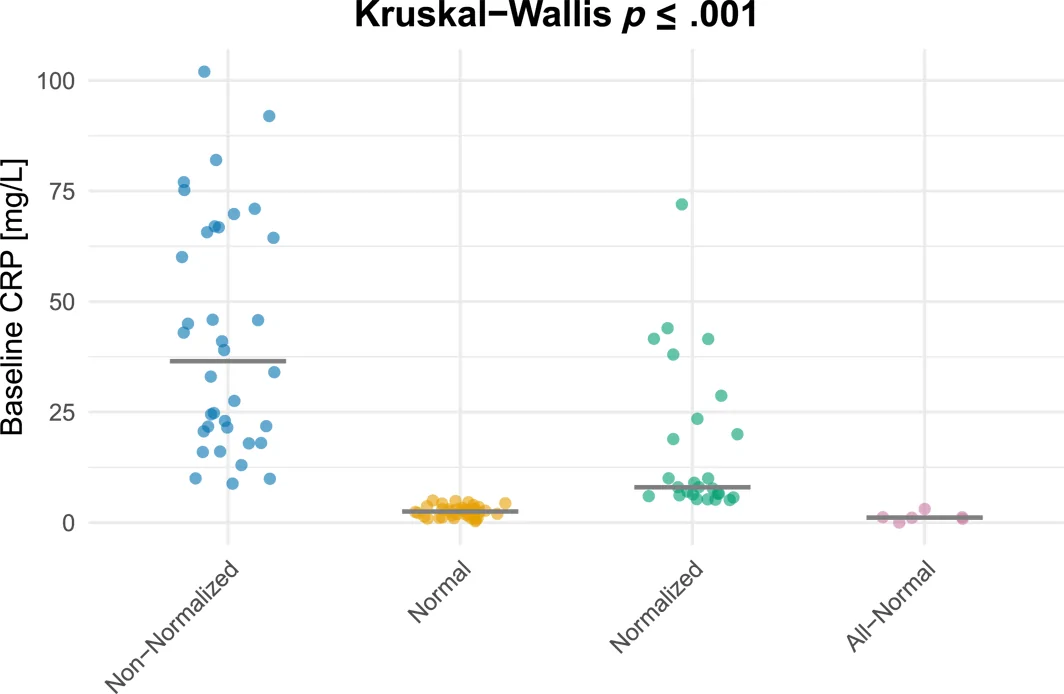
Comparison of median baseline C‐reactive protein levels in the refined model. CRP indicates C‐reactive protein.

**TABLE 4:** Comparison of subgroups according to the refined model of Ishihara et al.

Variables	No. (%)				*p*
	Non‐normalized, *n* = 36	Normal, *n* = 36	Normalized, *n* = 26	All‐normal, *n* = 6	
Age: Median [IQR], years	63 [43–83]	62 [27–77]	61 [38–84]	65 [42–76]	.899
Female	13 (36.1)	17 (47.2)	12 (46.2)	4 (66.7)	.502
Subtype of biliary tract cancer					.27
Intrahepatic cholangiocarcinoma	23 (63.9)	17 (47.2)	17 (65.4)	2 (33.3)	
Extrahepatic cholangiocarcinoma	12 (33.3)	16 (44.4)	6 (23.1)	4 (66.7)	
Gallbladder carcinoma	1 (2.8)	3 (8.3)	3 (11.5)	0 (0.0)	
Metastatic disease	26 (72.2)	24 (66.7)	16 (61.5)	3 (50.0)	.672
Greatest lesion dimension [IQR] in cm, *n* = 74	7.4 [3.9–10.7]	2.6 [1.4–6.2]	3.9 [2.5–7.5]	2.3 [1.0–17.7]	.005^a^
Known history of viral hepatitis	1 (2.8)	5 (13.9)	1 (3.8)	0 (0.0)	.203
Molecular profiling, *n* = 8					
MSI high	1 (3.7)	1(3.3)	0 (0.0)	1 (20.0)	.199
FGFR2	3 (11.1)	3 (10.0)	1 (4.8)	0 (0.0)	.759
IDH1	1 (3.7)	4 (13.3)	3 (14.3)	0 (0.0)	.455
BRAF	2 (7.4)	0 (0.0)	1 (4.8)	0 (0.0)	.474
ECOG PS >0, *n* = 102	17 (47.2)	8 (22.9)	13 (52.0)	1 (16.7)	.048^a^
Concurrent steroid medication	2 (5.6)	0 (0.0)	2 (7.7)	0 (0.0)	.401
Time to first staging: Median [IQR], days	73 [61–88]	78 [62–96]	76 [62–104]	77 [50–83]	.748
Time between stagings in days: Median [IQR], *n* = 33	91 [81–97]	82 [76–102]	92 [82–109]	75 [73–77]	.086
Laboratory parameters [range]					
Baseline CRP, mg/L	36.5 [8.8–102]	2.5 [0.3–4.9]	8 [5.1–72]	1.2 [0–3.1]	< .001^a^
Albumin, g/L	37 [20–47]	41 [31–48]	39 (31–47]	38 [34–44]	.009^a^
Bilirubin, mg/dl	0.4 [0.3–1.3]	0.5 [0.3–0.8]	0.4 [0.3–0.6]	0.7 [0.5–1.0]	.58
NLR	5.4 [0.19–16.14]	2.3 [0.01–7.45]	2.9 [0.1–7.9]	1.5 [0.33–7.2]	.022^a^
CA 19‐9 kU/L	136 [2–51,112]	32 [2–5529]	92 [2–50,000]	1191 [23–63,719]	.011^a^

Abbreviations: CRP, C‐reactive protein; CA 19‐9, carbohydrate antigen 19‐9; ECOG PS, Eastern Cooperative Oncology Group performance status; FGFR2, fibroblast growth factor receptor 2; IDH1, isocitrate dehydrogenase 1; IQR, interquartile range; MSI, microsatellite instability; NLR, neutrophil to lymphocyte ratio.

^a^ These *p* values indicate statistical significance.

Both univariable and multivariable Cox regression analyses indicated that the new CRP response model was significantly associated with PFS and OS (Tables 2 and 3). The median PFS differed significantly between the groups (*p* < .001) and was *not reached* (95% CI, not reached to not reached) for the all‐normal group, 11.0 months (95% CI, 6.7–15.3 months) for the normal group, 6 months (95% CI, 3.6–8.4 months) for the normalized group, and 2.0 months (95% CI, 0.9–3.1 months) for the non‐normalized group (Figure 3A). The median OS also differed significantly between the groups (*p* = .005) and was not reached (95% CI, not reached to not reached) for the all‐normal group, 26 months (95% CI, 15.5–36.5 months) for the normal group, 23 months (95% CI, 12.0–34.0 months) for the normalized group, and 9.0 months (95% CI, 6.3–11.7 months) for the non‐normalized group (Figure 3B); the DCRs were 100%, 77.8%, 60%, and 48.5%, respectively, and the ORRs were 50%, 33.3%, 36.0%, and 21.2%, respectively.

**FIGURE 3:**
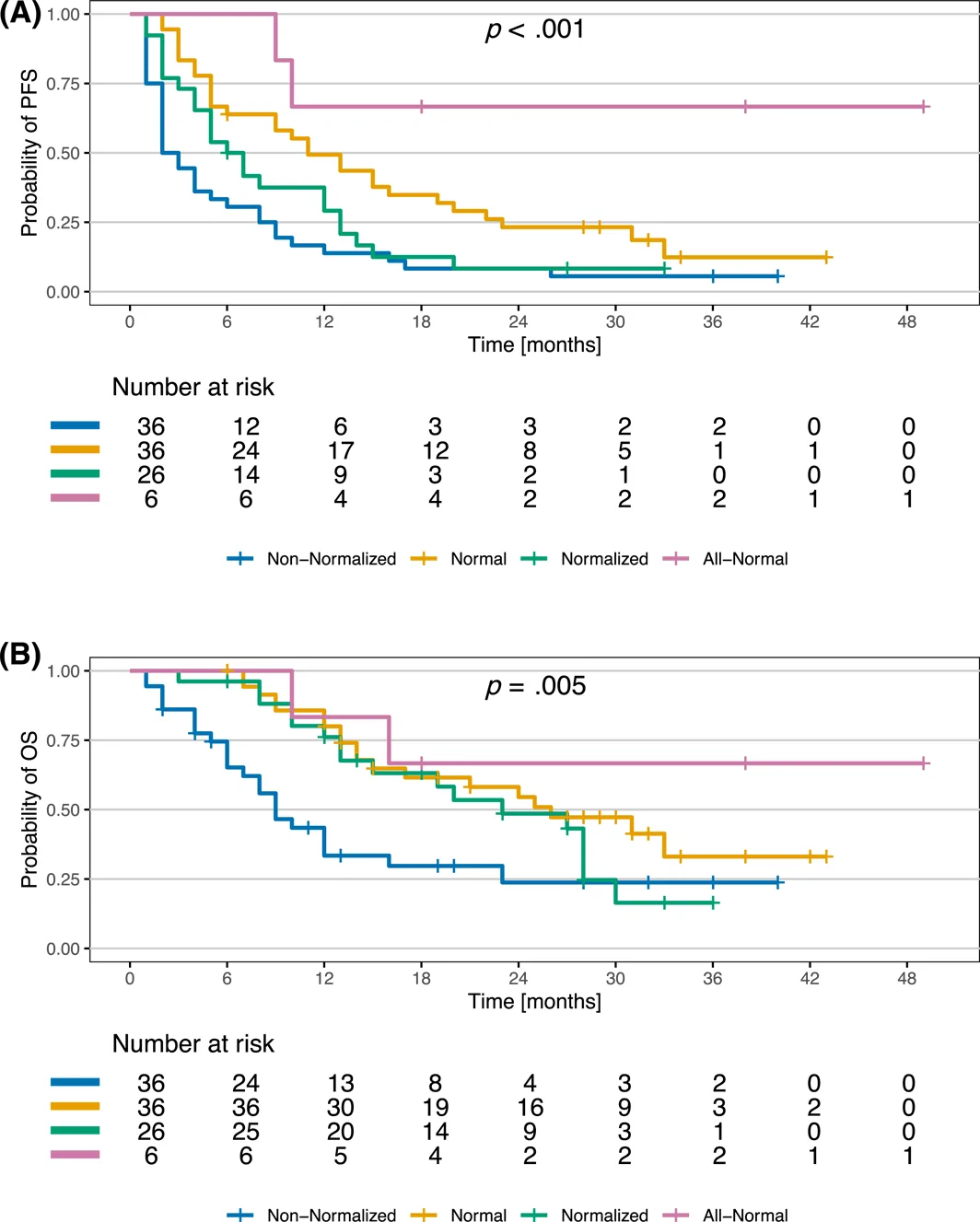
Kaplan–Meier plots for (A) overall survival and (B) progression‐free survival according to the refined CRP kinetics model. CRP indicates C‐reactive protein; OS, overall survival; PFS, progression‐free survival.

To address possible immortal time bias, landmark analysis was performed. The associations of the original Ishihara et al. model and our modified classification with PFS and OS remained significant after the 6‐week landmark analysis (see Figure S4A–D). In the 3‐month landmark analysis, the association with OS prevailed, whereas the association with PFS was attenuated (see Figure S3A–D).

### Six‐week adjustment

Although the initial definition of CRP kinetics by Ishihara et al. allows for the classification of patients as normalized or non‐normalized by the time of the first radiologic assessment at 3 months, the individual CRP dynamics suggest that more nuanced criteria may enable earlier distinction between these groups (Figure 4). Notably, 87.8% of patients in the normalized CRP group could be accurately classified as early as 6 weeks after treatment initiation. Among patients with initial CRP values below the ULN, 30.1% maintained CRP levels consistently below the ULN during the first 6 weeks and thus would be reclassified into the *normalized* group under the 6‐week model (see Figure S2).

**FIGURE 4:**
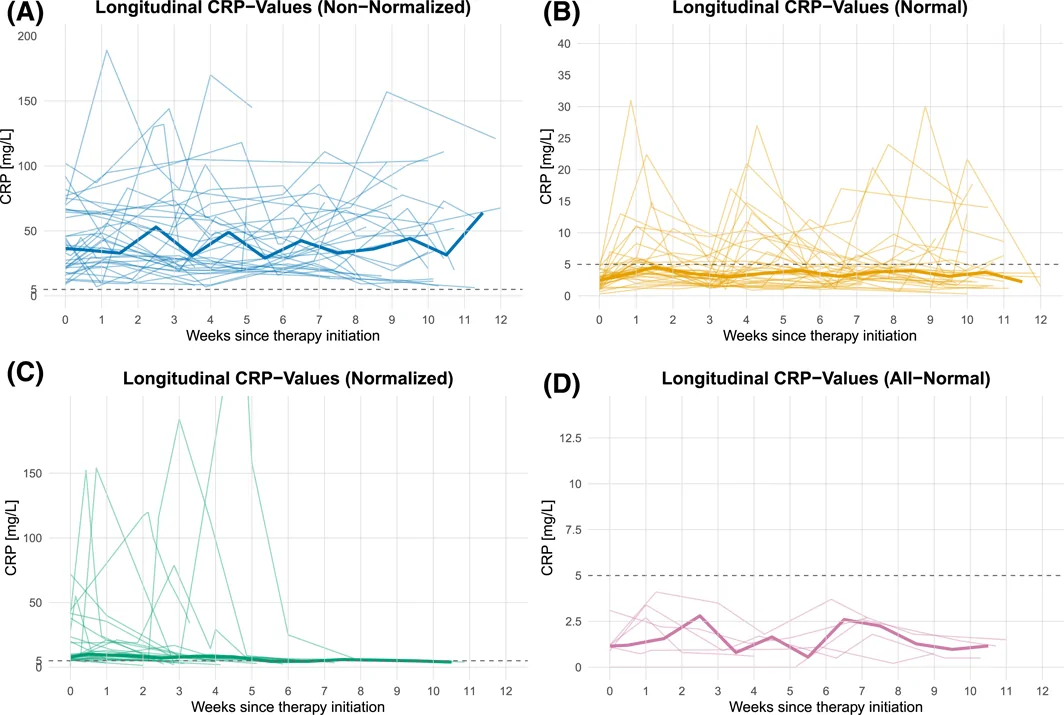
C‐reactive protein kinetics for all patients in the subgroups. Lines are censored after drop of CRP values below 5 mg/dL in the *normalized* graph for better visualization. CRP indicates C‐reactive protein.

The prognostic ability of survival by the revised model remained consistent when the observation period was reduced to 6 weeks. The median PFS differed significantly across the groups (*p* < .001), with estimates of 23 months (95% CI, 0.0–51.4 months) in the all‐normal group, 10.0 months (95% CI, 3.2–16.9 months) in the normal group, 7.0 months (95% CI, 4.0–10.0 months) in the normalized group, and 3.0 months (95% CI, 1.8–4.2 months) in the non‐normalized group (Figure 5A). Similarly, the median OS maintained significant differences between groups (*p* = .002), with a median of 31 months (95% CI, not reached to not reached) for the all‐normal group, 24 months (95% CI, 13.4–34.6 months) for the normal group, 27 months (95% CI, 21.4–32.6 months) for the normalized group, and 10.0 months (95% CI, 7.7–12.3 months) for the non‐normalized group (Figure 5B);the DCRs were 94.1%, 72.0%, 61.9%, and 48.6%, respectively (*p* = .01), and the ORRs were 29.4%, 40.0%, 42.9%, and 18.9% (*p* = .181), respectively.

**FIGURE 5:**
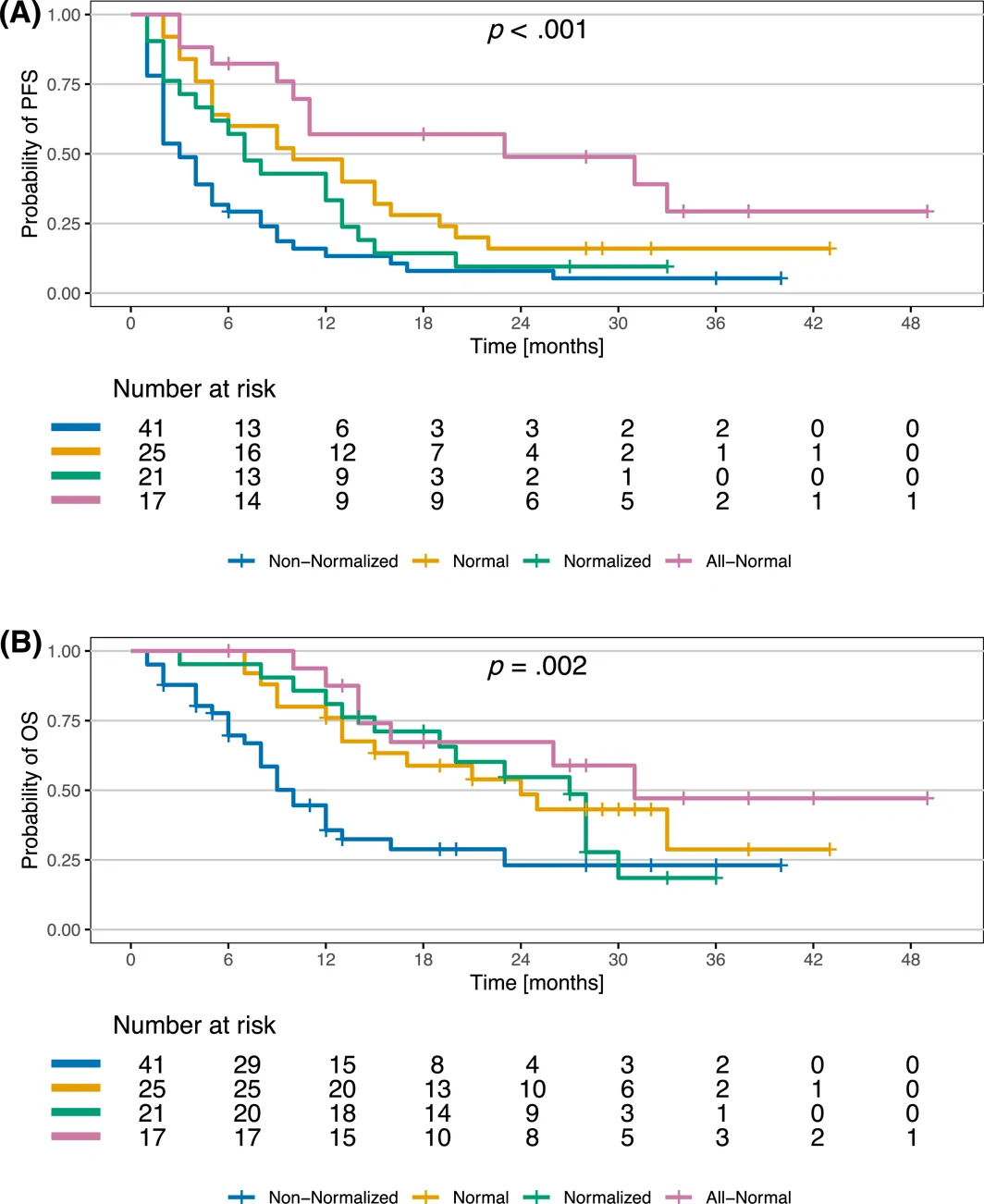
Kaplan–Meier plots for (A) progression‐free survival and (B) overall survival according to our refined CRP kinetics model with the 6‐week cutoff. CRP indicates C‐reactive protein; OS, overall survival; PFS, progression‐free survival.

## DISCUSSION

The results of this national, multicenter study demonstrate the prognostic value of early CRP kinetics for survival outcomes in patients with BTC who receive chemoimmunotherapy. Although the Fukuda et al. model did not demonstrate prognostic value in this cohort, the model of Ishihara et al. was significantly associated with survival. It is noteworthy that the prognostic ability of Ishihara and colleagues’ model maintained significance as early as within the first 6 weeks after treatment initiation. Thus longitudinal, on‐treatment measurement of CRP levels may represent a noninvasive and cost‐effective biomarker for individualized treatment monitoring.

Inflammation plays an important role in angiogenesis, proliferation, as well as tumor immunosuppression and is associated with elevated CRP levels.^26^ CRP is also associated with advanced cancer stages and a poor prognosis in pan‐agnostic cancer studies and has also been correlated with resistance to certain chemotherapies.^27^ Therefore, decreasing or sustained levels of CRP below the ULN during systemic therapy may hint at mild inflammation and beneficial tumor response and prognosis.

In earlier studies, baseline CRP levels were evaluated as a prognostic biomarker in liver cancer therapy^28^, ^29^; however, evidence of the prognostic performance of early CRP dynamics in patients with BTC who receive chemoimmunotherapy was insufficient before the initiation of our study. CRP kinetics were analyzed previously in other tumor entities treated with ICI monotherapy or in combination with targeted therapy.^18^, ^22^, ^23^, ^30^ However, in our study, we were able to demonstrate the significant prognostic value of the CRP classification model by Ishihara et al. in patients with BTC who received ICIs in combination with chemotherapy for the first time. It is interesting to note that, by refining the model, we were able to demonstrate that patients with initially normal CRP values, which increased during treatment (normal group), still experienced favorable outcomes compared with patients who had non‐normalized CRP levels. The newly defined subgroup all‐normal CRP comprised only about 6% of the initial population; however, the high DCRs and ORRs of this group were consistent in the larger all‐normal group in the 6‐week model. Subgroup analysis revealed that these patients had the lowest dimension of the greatest tumor lesion and the lowest baseline levels of CRP and NLR, which are favorable prognostic markers. However, patients in this group also had the highest CA 19‐9 levels, which are associated with an unfavorable outcome.^31^ Given the small sample size of this subgroup, these findings are hypothesis‐generating and require external, prospective validation in larger cohorts. We were also able to demonstrate that the subgroup of patients with elevated CRP values that normalized within 12 weeks of treatment had outcomes comparable to those of patients with initially normal CRP values. Therefore, sole reliance on the baseline CRP measurement appears insufficient for reliable prediction of survival.

The prognostic performance of our refined model remained consistent even when the observation period was limited to 6 weeks. Because CRP levels normalized within this timeframe in 87.8% of patients in the originally defined normalized subgroup, it may allow earlier identification of patients who are unlikely to benefit from chemoimmunotherapy. The identification of nonresponders may support clinical decision‐making and may facilitate timely transition to promising second‐line treatment regimens, such as targeted therapy, before the deterioration of organ function and performance status occur.

Fukuda et al. defined three groups of patients with renal cell carcinoma based on their longitudinal changes of CRP values in reference to baseline levels within the first 3 months after ICI initiation. Those authors demonstrated that patients who had a CRP flare exhibited significantly longer PFS and OS and higher ORRs than CRP nonresponders. Recently, this prognostic model has been validated between other tumor entities like non–small cell lung cancer, urothelial carcinoma and, melanoma.^19^, ^21^, ^22^ Furthermore, Klumper et al. demonstrated that approximately 90% of flare responders could be identified within the first 4–6 weeks after ICI treatment initiation.^19^, ^30^ In a recent study, Barth et al. further refined the CRP kinetics groups by identifying the prognostic significance of persistently normal low CRP values below the ULN.^22^ In their study, patients with all‐normal CRP exhibited the most favorable ORRs and survival rates, similar to the results of our refined model.

In our study, the flare‐response model by Fukuda et al. failed to reliably predict response and survival in patients who received chemoimmunotherapy. These results are comparable to a prior study by Klumper et al. in which this CRP kinetics model also failed to predict relevant outcomes if concomitant chemotherapy was applied.^19^ The prognostic value of a CRP flare response may be attenuated by concurrent chemotherapy because chemotherapy itself is cytotoxic and induces tissue injury as well as systemic inflammation, which can be accompanied by CRP elevation. Thereby, the specific CRP dynamics of ICI therapy might be obscured and the prognostic ability of the flare‐response model diminished.

In our univariate and multivariate analyses, other inflammation parameters, such as NLR and albumin levels, were not associated with PFS and OS. Prior studies hint at an association of low NLRs and a beneficial response to ICI therapy; however, the recent TOPAZ‐1 and KEYNOTE‐966 studies also could not demonstrate an association in patients who received chemoimmunotherapy.^6^, ^8^

Although it identifies a valuable prognostic biomarker for ICI treatment in BTC in a national multicenter study, this study certainly has some limitations. Naturally, the retrospective design of our study carries the risk of selection bias. However, we accounted for the possible immortal time bias by using a landmark analysis, which largely confirmed the prognostic value of the kinetic models. However, in the 3‐month landmark analysis, the association with PFS was no longer statistically significant for either the Ishihara et al. model (*p* = .082) or the modified model (*p* = .078), whereas the association with OS remained significant. Furthermore, subclinical or occult infections leading to elevated CRP levels may not always have been identified and, consequently, may have been excluded. Consequently, patients with otherwise normal or normalized CRP values might experience an infection‐related rise above the ULN and thus could be misclassified as having non‐normalized CRP. Although this potential source of bias cannot be fully eliminated, it is noteworthy that not only did survival‐based end points differ in our analysis, but tumor‐related end points, such as DCR and, by trend, ORR, also differed. The consistency of these tumor‐specific outcomes makes a systematic misinterpretation of survival effects caused by insufficiently recognized infections less likely, yet this limitation should still be considered when interpreting the robustness of early CRP kinetics as a prognostic marker in everyday practice. In addition, some short CRP flares may have been missed between two CRP measurements because no uniform, strict protocol of measurement timing existed. CRP measurement routines varied substantially across participating centers, with certain centers omitting testing unless infection was suspected, potentially contributing to the high proportion of patients in the overall cohort who failed to meet the inclusion criteria. Although CRP values during treatment of this rare tumor type were not always routinely collected, our cohort is relatively large compared with cohorts from other studies on CRP kinetics or durvalumab treatment in BTC.

## CONCLUSION

CRP kinetics may be a valuable prognostic tool for early outcome assessment in patients with BTC receiving chemoimmunotherapy and could be applied as early as within 6 weeks after treatment initiation. In particular, non‐normalized CRP values were associated with unfavorable outcomes. Our findings warrant further prospective evaluation in larger cohorts to determine whether CRP‐guided treatment adaptation improves clinical outcomes.

## AUTHOR CONTRIBUTIONS

**Gabriel Allo**: Conceptualization; investigation; writing–original draft; visualization; methodology; writing–review and editing; formal analysis; data curation. **Joshua Leutiger**: Conceptualization; data curation; formal analysis; visualization; writing–review and editing. **Catherine Leyh**: Writing–review and editing; data curation; investigation. **Christian Mohring**: Investigation; data curation; writing–review and editing. **Ann‐Kristin Zoller**: Data curation; writing–review and editing; investigation. **Vera Himmelsbach**: Investigation; writing–review and editing; data curation. **Gianluca Ciarlo**: Investigation; writing–review and editing; data curation. **Maryam Barsch**: Investigation; writing–review and editing; data curation. **Christoph Neumann‐Haefelin**: Investigation; conceptualization; validation; methodology; writing–review and editing; project administration; supervision. **Bertram Bengsch**: Supervision; conceptualization; writing–review and editing; validation. **Fabian Finkelmeier**: Writing–review and editing; conceptualization; supervision; validation. **Mohamad Murad**: Investigation; writing–review and editing; data curation. **Cennet Sahin**: Investigation; writing–review and editing; data curation. **Marie‐Luise Berres**: Writing–review and editing; validation; supervision. **Maria A. Gonzalez‐Carmona**: Conceptualization; writing–review and editing; validation; supervision. **Christoph Roderburg**: Supervision; writing–review and editing; validation; data curation; formal analysis; investigation. **Arndt Weinmann**: Writing–review and editing; data curation; validation; investigation. **Anna Saborowski**: Conceptualization; writing–review and editing; supervision; validation. **Arndt Vogel**: Validation; conceptualization; writing–review and editing; supervision. **Dirk Waldschmidt**: Conceptualization; investigation; writing–review and editing; validation; methodology; formal analysis; project administration; supervision.

## CONFLICT OF INTEREST STATEMENT

Gabriel Allo reports support for professional activities from Bristol Myers Squibb Company and formerly owned stock in Curevac, Emergent Biosolutions, and Bavarian Nordic outside the submitted work. Ann‐Kristin Zoller owns stock in Novo Nordisk and Vertex Pharmaceuticals outside the submitted work. Maryam Barsch reports personal/consulting fees from Taiho Oncology; and support for other professional services from Astellas Pharma and the Falk Foundation outside the submitted work. Christoph Newmann‐Haefelin reports grants/contracts, speaking fees, and travel support from AbbVie Deutschland, the Falk Foundation, and Gilead Sciences Inc. outside the submitted work. Bertram Bengsch reports personal/consulting fees or honoraria from AstraZeneca, Bristol Myers Squibb Company, and F. Hoffmann‐La Roche AG outside the submitted work and has a family member who is an employee of Falk Pharma. Fabian Finkelmeier reports personal/consulting fees from AbbVie, AstraZeneca, Bristol Myers Squibb Company, Merck Sharp & Dohme, F. Hoffman‐La Roche, and Servier; and travel support from AbbVie, AstraZeneca, Bristol Myers Squibb Company, F. Hoffman‐La Roche, Merck Sharp & Dohme, and Servier outside the submitted work. Marie‐Luise‐Berres reports advisory fees from AstraZeneca, Bayer, Bristol Myers Squibb Company, Daiichi‐Sankyo Company, EISAI Inc., F. Hoffmann‐La Roche, and Merck Sharp & Dohme; speaking fees from Astellas, AstraZeneca, Bristol Myers Squibb Company, Eli Lilly and Company, F. Hoffman‐La Roche, Merck Sharp & Dohme, Pierre Fabre Pharmaceuticals Inc., and Servier; and travel support from AstraZeneca and F. Hoffman‐La Roche outside the submitted work. Maria A. Gonzalez‐Carmona reports research funding from EISAI Inc.; personal/consulting fees from Amgen, AstraZeneca, Bristol Myers Squibb Company, Eli Lilly and Company, F. Hoffman‐La Roche, Merck Sharp & Dohme, and Servier; and travel support from Amgen, AstraZeneca, Bristol Myers Squibb Company, Daiichi‐Sankyo Company, and Servier outside the submitted work. Christoph Roderberg reports advisory fees from AstraZeneca, Bristol Myers Squibb Company, EISAI Inc., F. Hoffmann‐La Roche, Incyte, Merck Sharp & Dohme, Servier, and Taiho Oncology; speaking fees from AstraZeneca, Boehringer Ingelheim, Bristol Myers Squibb Company, EISAI Inc., F. Hoffman‐La Roche, Merck Sharp & Dohme, Servier, and Taiho Oncology; and travel support from AstraZeneca, F. Hoffmann‐La Roche, Ipsen, and Servier outside the submitted work. Anna Saborowski reports personal/consulting fees from Bristol Myers Squibb Company, EISAI Inc., Merck Sharp & Dohme, Incyte Corporation, and Roche; honoraria from Amgen, AstraZeneca, Bristol Myers Squibb Company, EISAI Inc., Eli Lilly and Company, Ipsen, Merck Sharp & Dohme, Roche, and Servier; and travel support from EISAI Inc., Merck Sharp & Dohme, Pierre Fabre Pharmaceuticals Inc., and Servier outside the submitted work. Arndt Vogel reports research funding from Novartis; personal/consulting fees or honoraria from AbbVie, AstraZeneca, Baxalta. BeiGene, Biologix, Bohringer Pharma GmbH, Boston Scientific Corporation, Bristol Myers Squibb Company, BTG, Daiichi‐Sankyo Company, EISAI Inc., Elevar, F. Hoffmann‐La Roche, GlaxoSmithKline, Incyte/Medimmune, Ipsen Bioscience Inc., Janssen Global Services LLC, Jazz Pharmaceuticals, Lilly Deutschland, Merck Sharp & Dohme, Novartis, Pierre Fabre Pharmaceuticals Inc., Servier, Sirtex Medical Inc., Tahio Oncology, Terumo, Tyra Biosciences, and ZymeWorks; travel support from Eli Lilly and Company, F. Hoffmann‐La Roche, Ipsen, and Merck Sharp & Dohme; and support for other professional activities from MD Health, OncLive, Oncowissen, and Research to Practice outside the submitted work. Dirk Waldschmidt reports advisory fees from AstraZeneca, Bristol Myers Squibb Company, EISAI Inc., F. Hoffmann‐La Roche, Incyte, Merck Sharp & Dohme, Servier, and Taiho Oncology; payment or honoraria for lectures, presentations, speakers’ bureaus, article writing, or educational events from AstraZeneca, Boehringer Ingelheim, EISAI Inc., F. Hoffmann‐La Roche, Incyte, Merck Sharp & Dohme, Servier, and Sirtex; payment for expert testimony from AstraZeneca, Bristol Myers Squibb Company, EISAI Inc., F. Hoffmann‐La Roche, Incyte, Merck Sharp & Dohme, Servier, and Taiho Oncology; and travel/meeting support from AstraZeneca, Ipsen, Roche, and Servier outside the submitted work. The remaining authors disclosed no conflicts of interest.

## Supporting information

Supporting Information S1

Figure S1

Figure S2

Figure S3

Figure S4

Table S1

Table S2

## Data Availability

The data that support the findings of this study are available on request from the corresponding author. The data are not publicly available because of privacy or ethical restrictions.

## References

[1] Tamandl D , Herberger B , Gruenberger B , Puhalla H , Klinger M , Gruenberger T . Influence of hepatic resection margin on recurrence and survival in intrahepatic cholangiocarcinoma. Ann Surg Oncol. 2008;15(10):2787‐2794. doi:10.1245/s10434-008-0081-1 18685896

[2] Wang Y , Li J , Xia Y , et al. Prognostic nomogram for intrahepatic cholangiocarcinoma after partial hepatectomy. J Clin Oncol. 2013;31(9):1188‐1195. doi:10.1200/JCO.2012.41.5984 23358969

[3] Matsuo K , Rocha FG , Ito K , et al. The Blumgart preoperative staging system for hilar cholangiocarcinoma: analysis of resectability and outcomes in 380 patients. J Am Coll Surg. 2012;215(3):343‐355. doi:10.1016/j.jamcollsurg.2012.05.025 22749003

[4] Fong Y , Jarnagin W , Blumgart LH . Gallbladder cancer: comparison of patients presenting initially for definitive operation with those presenting after prior noncurative intervention. Ann Surg. 2000;232(4):557‐569. doi:10.1097/00000658-200010000-00011 PMC1421188 10998654

[5] Valle J , Wasan H , Palmer DH , et al. Cisplatin plus gemcitabine versus gemcitabine for biliary tract cancer. N Engl J Med. 2010;362(14):1273‐1281. doi:10.1056/NEJMoa0908721 20375404

[6] Oh DY , Ruth He A , Qin S , et al. Durvalumab plus gemcitabine and cisplatin in advanced biliary tract cancer. NEJM Evid. 2022;1(8):EVIDoa2200015. doi:10.1056/EVIDoa2200015 38319896

[7] Oh DY , He AR , Bouattour M , et al. Durvalumab or placebo plus gemcitabine and cisplatin in participants with advanced biliary tract cancer (TOPAZ‐1): updated overall survival from a randomised phase 3 study. Lancet Gastroenterol Hepatol. 2024;9(8):694‐704. doi:10.1016/S2468-1253(24)00095-5 38823398

[8] Kelley RK , Ueno M , Yoo C , et al. Pembrolizumab in combination with gemcitabine and cisplatin compared with gemcitabine and cisplatin alone for patients with advanced biliary tract cancer (KEYNOTE‐966): a randomised, double‐blind, placebo‐controlled, phase 3 trial. Lancet. 2023;401(10391):1853‐1865. doi:10.1016/S0140-6736(23)00727-4 37075781

[9] McGrail DJ , Pilié PG , Rashid NU , et al. High tumor mutation burden fails to predict immune checkpoint blockade response across all cancer types. Ann Oncol. 2021;32(5):661‐672. doi:10.1016/j.annonc.2021.02.006 PMC8053682 33736924

[10] Marabelle A , Fakih M , Lopez J , et al. Association of tumour mutational burden with outcomes in patients with advanced solid tumours treated with pembrolizumab: prospective biomarker analysis of the multicohort, open‐label, phase 2 KEYNOTE‐158 study. Lancet Oncol. 2020;21(10):1353‐1365. doi:10.1016/S1470-2045(20)30445-9 32919526

[11] Goeppert B , Roessler S , Renner M , et al. Mismatch repair deficiency is a rare but putative therapeutically relevant finding in non‐liver fluke associated cholangiocarcinoma. Br J Cancer. 2019;120(1):109‐114. doi:10.1038/s41416-018-0199-2 PMC6325153 30377340

[12] Mitzlaff K , Kirstein MM , Müller C , et al. Efficacy, safety and differential outcomes of immune‐chemotherapy with gemcitabine, cisplatin and durvalumab in patients with biliary tract cancers: a multicenter real world cohort. United European Gastroenterol J. 2024;12(9):1230‐1242. doi:10.1002/ueg2.12656 PMC11578849 39301763

[13] Goyal L , Meric‐Bernstam F , Hollebecque A , et al. Futibatinib for *FGFR2*‐rearranged intrahepatic cholangiocarcinoma. N Engl J Med. 2023;388(3):228‐239. doi:10.1056/NEJMoa2206834 36652354

[14] Abou‐Alfa GK , Macarulla T , Javle MM , et al. Ivosidenib in IDH1‐mutant, chemotherapy‐refractory cholangiocarcinoma (ClarIDHy): a multicentre, randomised, double‐blind, placebo‐controlled, phase 3 study. Lancet Oncol. 2020;21(6):796‐807. doi:10.1016/S1470-2045(20)30157-1 PMC7523268 32416072

[15] Abou‐Alfa GK , Sahai V , Hollebecque A , et al. Pemigatinib for previously treated, locally advanced or metastatic cholangiocarcinoma: a multicentre, open‐label, phase 2 study. Lancet Oncol. 2020;21(5):671‐684. doi:10.1016/S1470-2045(20)30109-1 PMC8461541 32203698

[16] Kauffmann‐Guerrero D , Kahnert K , Kiefl R , et al. Systemic inflammation and pro‐inflammatory cytokine profile predict response to checkpoint inhibitor treatment in NSCLC: a prospective study. Sci Rep. 2021;11(1):10919. doi:10.1038/s41598-021-90397-y PMC8149421 34035415

[17] Fukuda S , Saito K , Yasuda Y , et al. Impact of C‐reactive protein flare‐response on oncological outcomes in patients with metastatic renal cell carcinoma treated with nivolumab. J Immunother Cancer. 2021;9(2):e001564. doi:10.1136/jitc-2020-001564 PMC7896625 33602695

[18] Klümper N , Schmucker P , Hahn O , et al. C‐reactive protein flare‐response predicts long‐term efficacy to first‐line anti–PD‐1‐based combination therapy in metastatic renal cell carcinoma. Clin Transl Immunol. 2021;10(12):e1358. doi:10.1002/cti2.1358 PMC8648498 34925829

[19] Klümper N , Saal J , Berner F , et al. C reactive protein flare predicts response to checkpoint inhibitor treatment in non–small cell lung cancer. J Immunother Cancer. 2022;10(3):e004024. doi:10.1136/jitc-2021-004024 PMC8928397 35292517

[20] Hoeh B , Garcia CC , Banek S , et al. Early CRP kinetics to predict long‐term efficacy of first‐line immune‐checkpoint inhibition combination therapies in metastatic renal cell carcinoma: an updated multicentre real‐world experience applying different CRP kinetics definitions. Clin Transl Immunology. 2023;12(10):e1471. doi:10.1002/cti2.1471 PMC10600333 37899949

[21] Klümper N , Sikic D , Saal J , et al. C‐reactive protein flare predicts response to anti‐PD‐(L)1 immune checkpoint blockade in metastatic urothelial carcinoma. Eur J Cancer. 2022;167:13‐22. doi:10.1016/j.ejca.2022.02.022 35366569

[22] Barth DA , Moik F , Steinlechner S , et al. Early kinetics of C reactive protein for cancer‐agnostic prediction of therapy response and mortality in patients treated with immune checkpoint inhibitors: a multicenter cohort study. J Immunother Cancer. 2023;11(12):e007765. doi:10.1136/jitc-2023-007765 PMC10729183 38097343

[23] Ishihara H , Takagi T , Kondo T , et al. Predictive impact of an early change in serum C‐reactive protein levels in nivolumab therapy for metastatic renal cell carcinoma. Urol Oncol. 2020;38(5):526‐532. doi:10.1016/j.urolonc.2019.12.008 31864934

[24] Eisenhauer EA , Therasse P , Bogaerts J , et al. New Response Evaluation Criteria in Solid Tumours: revised RECIST guideline (version 1.1). Eur J Cancer. 2009;45(2):228‐247. doi:10.1016/j.ejca.2008.10.026 19097774

[25] Allo G , Leutiger J , Leyh C , et al. Evaluation of early C‐reactive protein kinetics as an early response marker in biliary tract cancer patients treated with combined chemoimmunotherapy [abstract]. J Clin Oncol. 2026;44(2 suppl):530. doi:10.1200/JCO.2026.44.2_suppl.530 PMC13662409 42870461

[26] Rosenberg SA . Progress in human tumour immunology and immunotherapy. Nature. 2001;411(6835):380‐384. doi:10.1038/35077246 11357146

[27] Hart PC , Rajab IM , Alebraheem M , Potempa LA . C‐reactive protein and cancer—diagnostic and therapeutic insights. Front Immunol. 2020;11:595835. doi:10.3389/fimmu.2020.595835 PMC7727277 33324413

[28] Sprinzl MF , Kirstein MM , Koch S , et al. Improved prediction of survival by a risk factor‐integrating inflammatory score in sorafenib‐treated hepatocellular carcinoma. Liver Cancer. 2018;8(5):387‐402. doi:10.1159/000492628 PMC6873091 31768347

[29] Nakanishi H , Kurosaki M , Tsuchiya K , et al. Novel pretreatment scoring incorporating C‐reactive protein to predict overall survival in advanced hepatocellular carcinoma with sorafenib treatment. Liver Cancer. 2016;5(4):257‐268. doi:10.1159/000449337 PMC5075810 27781198

[30] Saal J , Bald T , Eckstein M , et al. Early C‐reactive protein kinetics predicts immunotherapy response in non–small cell lung cancer in the phase III OAK trial. JNCI Cancer Spectr. 2023;7(2):pkad027. doi:10.1093/jncics/pkad027 PMC10121335 37004206

[31] Grunnet M , Christensen IJ , Lassen U , et al. Decline in CA 19‐9 during chemotherapy predicts survival in four independent cohorts of patients with inoperable bile duct cancer. Eur J Cancer. 2015;51(11):1381‐1388. doi:10.1016/j.ejca.2015.04.011 25971532

