# Supplementary material for: Evaluation of early C‐reactive protein kinetics as an early response marker in biliary tract cancer treated with combined chemoimmunotherapy: A multicenter observational study
Source: Cancer. 2026 Oct 13;132(20):e70644. doi: 10.1002/cncr.70644 (PMC13662409; doi:10.1002/cncr.70644)
Supplement: Supplementary file 1 [file CNCR-132-e70644-s002.docx]

**Supplementary Table 1** Comparison of subgroups according to the model of Fukuda

| Variables | Flare responder (n=29) | Responder (n=43) | Non-responder (n=32) | p |
| --- | --- | --- | --- | --- |
| Age (years) (IQR) | 63 (61 – 68) | 63 (58 – 69) | 63 (56 – 70) | 0.086 |
| Female | 14 (48.3) | 16 (37.2) | 16 (50.0) | 0.476 |
| Subtype of biliary tract cancer |  |  |  | 0.209 |
| Intrahepatic cholangiocarcinoma | 13 (44.8) | 29 (67.4) | 17 (53.1) |  |
| Extrahepatic cholangiocarcinoma | 12 (41.4) | 13 (30.2) | 13 (40.6) |  |
| Gallbladder carcinoma | 4 (13.8) | 1 (2.3) | 2 (6.3) |  |
| Metastatic disease | 20 (69.0) | 26 (60.5) | 23 (71.9) | 0.551 |
| Largest lesion diameter (cm) (IQR) | 3.9 (1.9 – 7.9) | 5.7 (2.9 – 10.1) | 4.1 (1.8 – 7.0) | 0.159 |
| Known History of viral Hepatitis | 2 (6.9) | 2 (4.7) | 3 (9.4) | 0.721 |
| Molecular Profiling (n=83)  MSI high  FGFR2  IDH1  BRAF | 0  2 (9.5)  1 (4.8)  0 | 0  2 (6.3)  3 (9.4)  2 (6.3) | 3 (10.0)  3 (10.0)  4 (13.3)  1 (3.3) | 0.064  0.85  0.593  0.489 |
| ECOG >0 (n=102) | 12 (42.9) | 19 (44.2) | 8 (25.8) | 0.232 |
| Concurrent steroid medication | 1 (3.4) | 1 (2.3) | 2 (6.3) | 0.688 |
| Time to first staging (days) (IQR) | 72 (62 – 100) | 76 (59 – 89) | 81 (67 – 91) | 0.916 |
| Laboratory parameters |  |  |  |  |
| Baseline CRP | 2.2 (1.5 – 6.4) | 15 (5.2 – 41.5) | 3.4 (1.2 – 23.4) | **<0.001** |
| Albumin g/L | 40 (38 – 44) | 37 (34 – 40) | 41 (38 – 44) | **0.012** |
| NLR | 2.0 (0.4 – 3.0) | 3.3 (2.3 – 5.7) | 2.5 (2.0 – 5.2) | **0.048** |
| CA19-9 kU/L | 34 (10 – 214) | 110 (24 – 458) | 54 (15 – 257) | 0.758 |

Abbreviations: CRP= C-reactive protein; CA19-9= carbohydrate antigen 19‐9; ECOG= Eastern Cooperative Oncology Group; NLR= neutrophil to lymphocyte ratio
