# Supplementary material for: Evaluation of early C‐reactive protein kinetics as an early response marker in biliary tract cancer treated with combined chemoimmunotherapy: A multicenter observational study
Source: Cancer. 2026 Oct 13;132(20):e70644. doi: 10.1002/cncr.70644 (PMC13662409; doi:10.1002/cncr.70644)
Supplement: Supplementary file 2 [file CNCR-132-e70644-s004.docx]

**Supplementary materials**

**Figure Legends**

Supplementary Figure 1.	Patient Flow Chart

Supplementary Figure 2.	Sankey diagram of patients' flow between the refined Ishihara model and the six-week model

Supplementary Figure 3.	Kaplan-Meier plots and landmark analysis at three months for overall survival and progression-free survival according to the model of Ishihara (A= PFS, B=OS) and redefined model (C= PFS, D= OS)

Supplementary Figure 4.	Kaplan-Meier plots and landmark analysis at six weeks for overall survival and progression-free survival according to the model of Ishihara (A= PFS, B= OS) and redefined model (C= PFS, D =OS)
