# Supplementary material for: Evaluation of early C‐reactive protein kinetics as an early response marker in biliary tract cancer treated with combined chemoimmunotherapy: A multicenter observational study
Source: Cancer. 2026 Oct 13;132(20):e70644. doi: 10.1002/cncr.70644 (PMC13662409; doi:10.1002/cncr.70644)
Supplement: Supplementary file 3 [file CNCR-132-e70644-s007.docx]

**Supplementary Table 2** Comparison of subgroups according to the model of Ishihara

| Variables | CRP normal (n=42) | Normalized (n=26) | Non-normalized (n=36) | p |
| --- | --- | --- | --- | --- |
| Age (years) (IQR) | 63 (58 – 70) | 59 (54 – 64) | 64 (60 – 69) | 0.716 |
| Female | 21 (50.0) | 12 (46.2) | 13 (36.1) | 0.457 |
| Subtype of biliary tract cancer |  |  |  | 0.186 |
| Intrahepatic cholangiocarcinoma | 19 (45.2) | 17 (65.4) | 23 (63.9) |  |
| Extrahepatic cholangiocarcinoma | 20 (47.6) | 6 (23.1) | 12 (33.3) |  |
| Gallbladder carcinoma | 3 (7.1) | 3 (11.5) | 1 (2.8) |  |
| Metastatic disease | 27 (64.3) | 16 (61.5) | 26 (72.2) | 0.636 |
| Largest lesion diameter (cm) (IQR) (n=74) | 2.5 (1.4 – 6.3) | 3.9 (2.5 -7.5) | 7.3 (3.8 – 10.9) | **0.02** |
| Known History of viral Hepatitis | 5 (11.9) | 1 (3.8) | 1 (2.8) | 0.22 |
| Molecular Profiling (n=83)  MSI  FGFR2  IDH1  BRAF | 2 (5.7)  3 (8.6)  4 (11.4)  0 | 0  1 (4.8)  3 (14.3)  1 (4.8) | 1 (3.7)  3 (11.1)  1 (3.7)  2 (7.4) | 0.54  0.734  0.419  0.286 |
| ECOG <I | 32 (78.0) | 12 (48.0) | 19 (52.8) | 0.053 |
| Concurrent steroid medication | 0 | 2 (7.7) | 2 (5.6) | 0.23 |
| Time to first staging (days) (IQR) | 76 (65 – 91) | 77 (61 – 104) | 75 (61 – 89) | 0.785 |
| Time between stagings (days) (IQR) n=33 | 79 (75 – 99) | 92 (82 – 109) | 91 (78 -107) | 0.07 |
| Laboratory parameters |  |  |  |  |
| Baseline CRP | 2.0 (1.2 – 3.0) | 7.7 (6.4 – 28.7) | 42.0 (24.5 – 65.7) | **<0.001** |
| Albumin g/L | 41 (38 – 44) | 39 (34 – 41) | 37 (32 – 39) | **0.012** |
| Bilirubin (mg/dl) | 0.6 (0.3 – 0.8) | 0.4 (0.3 – 0.6) | 0.4 (0.3 – 1.2) | 0.577 |
| NLR | 2.3 (1.5 – 3.2) | 2.8 (0.6 – 3.2) | 5.4 (2.8 – 7.0) | **0.008** |
| CA19-9 kU/L | 32 (13 – 125) | 111 (24 – 308) | 179 (55 – 4425) | 0.088 |

Abbreviations: CRP= C-reactive protein; CA19-9= carbohydrate antigen 19‐9; ECOG= Eastern Cooperative Oncology Group; NLR= neutrophil to lymphocyte ratio
